# Single-Nucleotide Polymorphism of PPARγ, a Protein at the Crossroads of Physiological and Pathological Processes

**DOI:** 10.3390/ijms18020361

**Published:** 2017-02-10

**Authors:** Maria Petrosino, Laura Lori, Alessandra Pasquo, Clorinda Lori, Valerio Consalvi, Velia Minicozzi, Silvia Morante, Antonio Laghezza, Alessandra Giorgi, Davide Capelli, Roberta Chiaraluce

**Affiliations:** 1 Department of Biochemical Sciences “A. Rossi Fanelli”, Sapienza University of Rome, P.le A. Moro, 5, 00185 Rome, Italy; maria.petrosino@uniromad1.it (M.P.); laura.lori@uniroma1.it (L.L.); clorinda.lori@uniroma1.it (C.L.); valerio.consalvi@uniroma1.it (V.C.); alessandra.giorgi@uniroma1.it (A.G.); 2 SSPT-BIOAG-BIOTEC ENEA Casaccia ENEA, 00123 Rome, Italy; alessandra.pasquo@enea.it; 3 Department of Physics, University of Rome Tor Vergata and INFN, Via della Ricerca Scientifica 1, 00133 Roma, Italy; Velia.Minicozzi@roma2.infn.it (V.M.); silvia.morante@roma2.infn.it (S.M.); 4 Dipartimento di Farmacia-Scienze del Farmaco, University of Bari, 70126 Bari, Italy; antonio.laghezza@uniba.it; 5 Istituto di Cristallografia, Consiglio Nazionale delle Ricerche, Via Salaria Km. 29, 300, Monterotondo Stazione, 00015 Roma, Italy; davide.capelli80@gmail.com

**Keywords:** PPARγ, molecular dynamics, protein stability, single-nucleotide polymorphism

## Abstract

Genome polymorphisms are responsible for phenotypic differences between humans and for individual susceptibility to genetic diseases and therapeutic responses. Non-synonymous single-nucleotide polymorphisms (nsSNPs) lead to protein variants with a change in the amino acid sequence that may affect the structure and/or function of the protein and may be utilized as efficient structural and functional markers of association to complex diseases. This study is focused on nsSNP variants of the ligand binding domain of PPARγ a nuclear receptor in the superfamily of ligand inducible transcription factors that play an important role in regulating lipid metabolism and in several processes ranging from cellular differentiation and development to carcinogenesis. Here we selected nine nsSNPs variants of the PPARγ ligand binding domain, V290M, R357A, R397C, F360L, P467L, Q286P, R288H, E324K, and E460K, expressed in cancer tissues and/or associated with partial lipodystrophy and insulin resistance. The effects of a single amino acid change on the thermodynamic stability of PPARγ, its spectral properties, and molecular dynamics have been investigated. The nsSNPs PPARγ variants show alteration of dynamics and tertiary contacts that impair the correct reciprocal positioning of helices 3 and 12, crucially important for PPARγ functioning.

## 1. Introduction

**Figure 1:**
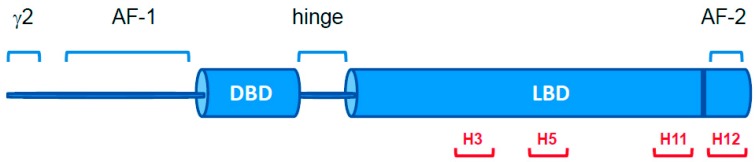
Schematic representation of nuclear receptor PPARγ. The ligand binding domain (LBD) is linked to the DNA binding domain (DBD) by a hinge. The residues involved in ligand binding are located in helix 3 (H3), helix 5 (H5), helix 11 (H11), and helix 12 (H12). Helices are numbered according to Nolte et al. [2]. PPARγ isoform 1 (UniProt ID P37231-2) is 28 residues shorter than PPARγ isoform 2 (UniProt ID P37231-1) at the N-terminus (γ2).

This study is focused on some natural variants of the Peroxisome Proliferator-Activated Receptor γ (PPARγ), a nuclear receptor that belongs to the superfamily of ligand inducible transcription factors, involved in several biological processes and in the maintenance of cellular homeostasis [1]. Nuclear receptors are multi-domain transcription factors that bind to DNA and regulate the expression of genes. PPARs (α, β/δ and γ) form heterodimers with retinoid X receptor (RXR) and, in the presence of a ligand, adopt an active conformation. Gene regulation by these receptors is related to the ligand-dependent recruitment of coactivators, which is necessary to create a complex that binds to Peroxisome Proliferator Response Elements (PPRE) [2,3]. PPARγ is composed of different functional domains: two activation functional domains, AF-1 and AF-2, and a ligand binding domain (LBD) connected to a DNA binding domain (DBD) by a hinge region (Figure 1).

PPARγ, expressed in the adipose tissue [4,5], regulates adipocyte differentiation and insulin sensitization, playing a key role in the regulation of lipid metabolism in mature adipocytes and macrophages [6], with a direct impact on type 2 diabetes, dyslipidemia, atherosclerosis, and cardiovascular diseases [7,8]. In addition to the role in lipid metabolism, PPARγ has been reported to play a role in several processes related to cellular differentiation and development and to carcinogenesis [9]. Moreover, PPARγ has been implicated in inflammation [10] and is expressed in colon, breast, and prostate cancers [4,9,11,12]. As far as the role played by PPARγ in cancer, the association of loss of function variants with colon cancer [13] along with some evidence of inhibition of cell proliferation and induction of apoptosis suggest its potential anti-neoplastic effects [9]. The activation of PPARγ by agonist drugs [14] such as thiazolidinediones has been proposed as antineoplastic therapy [15]. However, it is not yet clear whether the use of PPARγ ligands as drugs could reduce the risk of cancer development [15].

Some rare missense mutations in PPARγ may cause profound phenotypic changes in affected individuals, contributing to the risk of dyslipidemia, type 2 diabetes [16], and colon cancer [8,13,17,18,19,20,21,22,23]. Indeed, a point mutation in the PPARγ ligand-binding domain (LBD) may alter structural interactions that are important for its stabilization, thus affecting ligand binding and the receptor transcriptional function. The molecular mechanism of most PPARγ mutations, related to lipodystrophy and insulin resistance, is not clear [23,24] and the structural reason for the decrease in functional activity of PPARγ variants has been identified in the case of F360L [25] and V290M [26]. These variants are nsSNPs, or missense variants, i.e., single-nucleotide variations occurring in the DNA coding region that lead to a polypeptide with a change in the amino acid sequence. The effect of nsSNPs has been related to changes in protein stability, protein–protein interactions, and protein functions [27,28]. Comparative analyses of phenotypically vs. thermodynamically characterized variations revealed that, on average, the variation types most involved in disease are also associated with a pronounced effect on protein stability [29,30,31]. However, the strength of this association is not sufficient to consider protein destabilization as the unique mechanistic cause explaining the onset of diseases [29], and the impact of nsSNPs on protein function can be unambiguously clarified only by thorough experimental analysis [29,32]. Computational studies predicted that around 30% of protein variants resulting from nsSNPs are less stable than the wild type [33]. Moreover, in silico studies have predicted the impact of nsSNPs on protein structure, stability, function, and interactions and have analyzed how these variations may affect disease susceptibility [34,35]. However, the experimental assessment of in vitro stability of common variants is required to determine the biophysical effects of mutations on protein structure and function [32,36,37].

PPARγ, at the crossroads of physiological and pathological processes such as metabolic control and adipogenesis, inflammation, apoptosis, and cancer, is particularly interesting for the study of the effects of nsSNPs on its structural stability, thermodynamic, and dynamic properties in solution. Several natural variants of PPARγ LBD, such as F360L, V290M, R357A, R397C, and P467L, have been associated with lipid metabolism disorders as well as cancer, e.g., Q286P, R288H [13]. More than 30 PPARγ natural variants are reported in COSMIC, a database designed to store and display somatic mutation information relating to human cancers [38,39].

In this study we selected PPARγ variants V290M, R357A, R397C, F360L, P467L, Q286P, R288H, E324K, and E460K, all located in the LBD, which are expressed in cancer tissues and/or associated with partial lipodystrophy and insulin resistance. In the selection of the variants, we focused on those mutations that were located on putatively critical positions in the structure and that may lead to alteration of the polarity of the residue, such as E324K, E460K, R357A, and R397C, or in the secondary structure propensity, as in the case of Q286P. In particular, the variants Q286P, R288H, V290M, E324K, E460K, and P467L, located in H3, H5, H11, and H12, are in close proximity of the residues involved in ligand binding (Figures 1 and S1).

We have investigated the effect of single amino acid substitution on the thermal and thermodynamic stability and the spectral properties of the above mentioned PPARγ variants by comparing experimental data with molecular dynamics (MD) simulations.

The alterations in protein stability and function may be driven by non-covalent interactions changes and modification of conformational dynamics of the variants. In most cases the stability of the expressed protein variants has been suggested to be responsible for the impact and/or consequences of the mutations on the pathological conditions or genetic susceptibility to diseases of the individuals. However, recent studies on natural protein variants in solution revealed that the perturbation of tertiary structure is not necessarily followed by changes in thermal and/or thermodynamic stability [40]. Indeed, the changes in side-chain flexibility of a mutated residue may lead to local variation in protein dynamics. Analysis of physico-chemical properties of natural variants may be helpful to reveal local structural changes that may not affect the overall folding of the structure, or may not be evident from the analysis of the variants crystal structure due to the conformational constraints the protein is subjected to in the crystal [40].

MD simulations are well suited to capture effects of point mutations on protein dynamics and detect any minor changes associated with an nsSNP. Detailed knowledge at the atomic level allows for an understanding of the structural and functional relationship upon mutation. In this study we use MD (in silico) analysis and (in vitro) thermodynamic studies to investigate the effect of nsSNPs of PPARγ natural variants.

## 2. Results

### 2.1. PPARγ Variants

**Figure 2:**
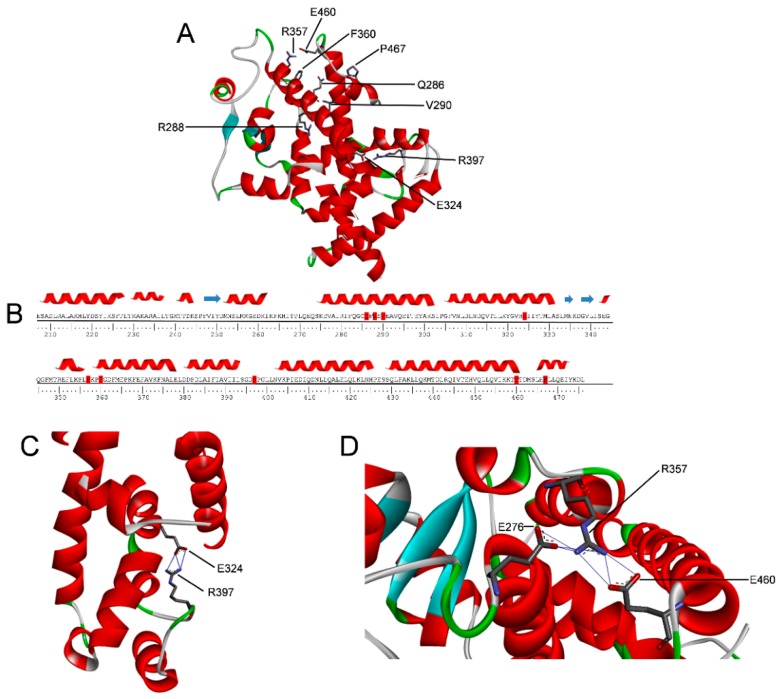
Amino acid sequence and structure of PPARγ LBD. (**A**) Structure of PPARγ LBD (PDB code: 1PRG) shown as a ribbon diagram; (**B**) secondary structural elements are shown at the top of the amino acid sequence. Mutated residues are highlighted in red; (**C**) Local environment of residues R397 and E324 involved in one salt bridge; (**D**) local environment of residue R357 engaging two salt bridges with residues E460 and E276.

In this study we focused on nine PPARγ variants (Q286P, R288H, V290M, E324K, R357A, F360L, R397C, E460K, and P467L) located in the LBD and associated with lipid metabolism disorders or to cancer (Figures 2 and S1). Four of these PPARγ variants, Q286P, R288H, E460K, and E324K have been found in cancer of the large intestine, lung, and endometrium, as reported in the COSMIC database (http://cancer.sanger.ac.uk/cosmic) [38]. The other five variants (V290M, R357A, F360L, P467L, and R397C) are related to alteration of metabolic control [23]. The variants Q286P, R288H, and V290M are located on helix 3 (H3), close to the ligand binding site, as is also the case with E324K, which is situated on helix 5 (H5). The variants R357A, E460K, and R397C are located in loops and F360L and P467L at the beginning of two small helices; the latter is the only variant in close proximity of one of the binding sites for the LXXLL helix of the coactivator [2]. The position of the mutated residues mapped onto the PPARγ structure is shown in Figures 2A and S1. The selected mutations encompass four surface exposed residues, R357A, R288H, E460K, and P467L, and five more buried residues, Q286P, V290M, F360L, R397C, and E324K. Site-directed mutagenesis and available bacterial expression systems were used to produce recombinant proteins of the identified mutants [25] with the purpose of studying the consequences of the mutations on PPARγ spectral properties and thermal and thermodynamic stability. Introduction of these mutations resulted in soluble recombinant protein for Q286P, R288H, V290M, R357A, F360L, E460K, and P467L, whereas E324K and R397C could not be expressed in the soluble fraction even when different induction conditions were used. Mapping of these mutations onto the structure of PPARγ LBD revealed that E324 and R397 are both involved in one of the two salt bridges that play a pivotal role in the domain stabilization (Figure 2C,D).

### 2.2. Spectroscopic Characterization of PPARγ Wild Type and Variants

**Figure 3:**
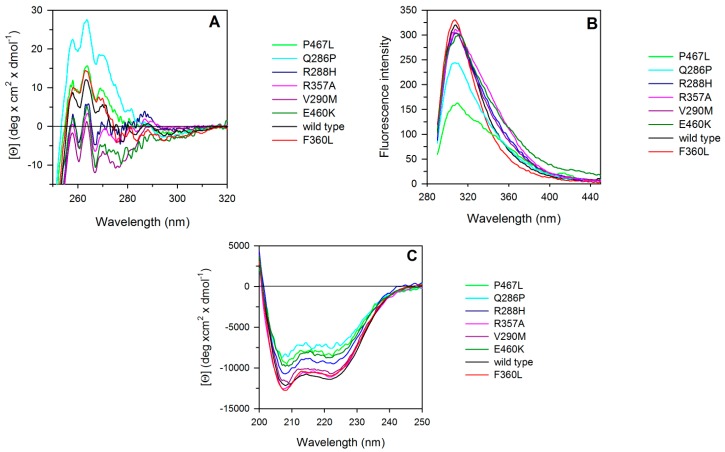
Spectroscopic properties of PPARγ wild type and variants. (**A**) Near-UV CD spectra were recorded in a 1 cm path-length quartz cuvette at 4.60 mg/mL protein concentration in 50 mM Tris–HCl pH 8.0 containing 0.20 M NaCl and 2.0 mM Dithiothreitol (DTT); (**B**) intrinsic fluorescence emission spectra were recorded at 0.1 mg/mL protein concentration (274 nm excitation wavelength) in 20 mM Tris–HCl pH 8.0 containing 0.1 M NaCl and 0.2 mM DTT; (**C**) far-UV CD spectra were recorded in a 0.1 cm path-length quartz cuvette at 0.2 mg/mL protein concentration in 20 mM Tris–HCl pH 8.0 containing 0.20 M NaCl and 0.2 mM DTT.

The near-UV circular dichroism (CD) spectrum of wild-type PPARγ, a protein lacking tryptophan residues, shows a strong positive contribution centered at around 263 nm, flanked by two positive shoulders at 270 nm and 258 nm, accompanied by fine structure features at 275–285 nm (Figure 3A). The near-UV CD spectra of F360L, P467L, and Q286P differ significantly from those of the wild type, either in intensity or in one of the positive shoulders that is blue-shifted to around 268 nm. In particular, the intensity of the near-UV CD spectrum of Q286P is significantly higher than that of the wild type and of F360L and P467L. V290M, R357A, R288H, and E460K display near-UV CD spectra closely similar to that of the wild type except for an overall decrease in the dichroic activity. Moreover, R357A and R288H show slight differences in the 270–280 nm region with respect to the wild type (Figure 3A).

The fluorescence emission spectra of the PPARγ wild type and variants are similar, but not identical. They all have a maximum emission wavelength around 308 nm, characteristic of tyrosine contribution (Figure 3B).

The far-UV CD spectra of PPARγ wild type and all the variants are typical of alpha helical proteins, showing local minima at around 208 and 222 nm and a zero intercept at around 200 nm. Interestingly, the wild type and variants show distinct contributions at 208 and 222 nm. The ratio of the molar ellipticity at 222 and at 208 nm ([Θ]_222_/[Θ]_208_) is 0.94 for the wild type and smaller for all the variants ranging from 0.86 for F360L, to 0.87 for R288H, 0.89 for R357A, 0.89 for E460K, 0.9 for P467L, 0.91 for Q296P, and 0.92 for V290M (Figure 3C). The 222/208 ellipticity ratio is indicative of interhelical contacts and has generally been used to distinguish between coiled coil helices and non-interacting helices (<0.9) [41,42]. The 222/208 ellipticity ratio below 0.9, observed for most of the variants, suggests different interhelical interactions and may indicate that the single amino acid substitutions induce significant changes of PPARγ structure in solution.

### 2.3. Thermal Unfolding

**Figure 4:**
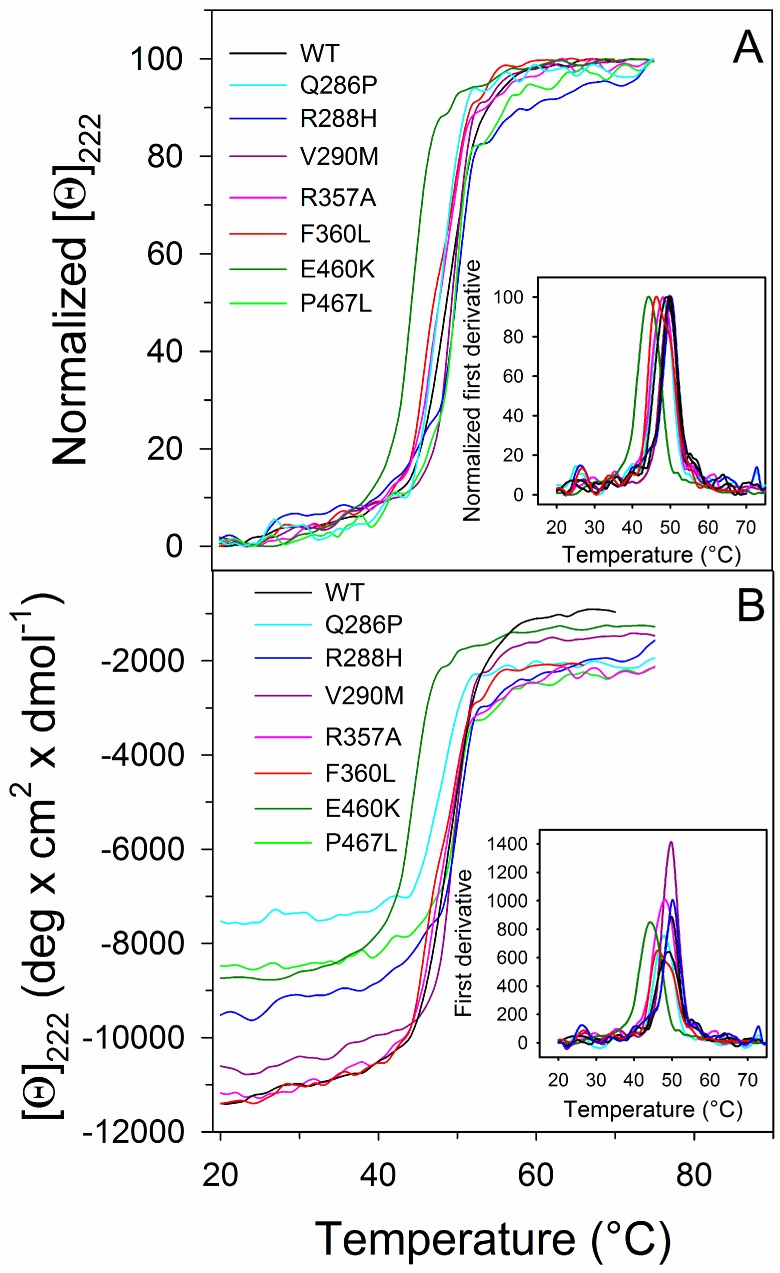
Thermal unfolding transition of PPARγ wild type and variants. Wild type and variants were heated from 20 to 75 °C in a 0.1 cm path-length quartz cuvette at 0.2 mg/mL protein in 20 mM Tris–HCl pH 8.0 containing 0.20 M NaCl and 0.2 mM DTT and the molar ellipticity at 222 nm ([Θ_222_]) was monitored continuously every 0.5 °C. (**A**) Normalized [Θ_222_]; (**B**) [Θ_222_] before normalization. The insets show the first derivative of the same data as in (**A**,**B**).

The thermal stability of PPARγ wild type and variants was investigated by continuously monitoring the ellipticity changes at 222 nm in the temperature range between 20 and 75 °C (Figure 4). The transition curves of PPARγ wild type and variants were compared by measuring the melting temperature (T_m_) that corresponds to the midpoint of the denaturation process as calculated by plotting the first derivative of the molar ellipticity values as a function of temperature (Figure 4 inset). The temperature-induced ellipticity changes at 222 nm, where the main amplitude was observed, occur in an apparent cooperative transition with T_m_ values ranging from 50.0 to 44.0 °C (Table 1). A modest increase in T_m_ values is observed for the variants P467L and R288H; all the other variants show T_m_ values lower than that of the wild type (Table 1), with E460K showing a T_m_ value five degrees below that of the wild type. Notably, the differences in the amplitude observed for the thermal transitions of most of the variants (Figure 4B) may be attributed to the difference in the dichroic activity at 222 nm of their corresponding native states, as also indicated in the far-UV CD spectra reported in Figure 3C. The ellipticity changes induced by temperature are paralleled by the increase of the photomultiplier tube voltage above 370 V (data not shown), suggesting that the protein aggregation follows temperature-induced unfolding. The observed transitions are irreversible, as indicated by the spectra measured at the end of the cooling phase that differ from those of the native proteins measured at the beginning of the thermal transitions. Furthermore, cuvette inspection at the end of the cooling phase revealed the presence of precipitate in all the samples.

### 2.4. Urea-Induced Equilibrium Unfolding Transitions

**Figure 5:**
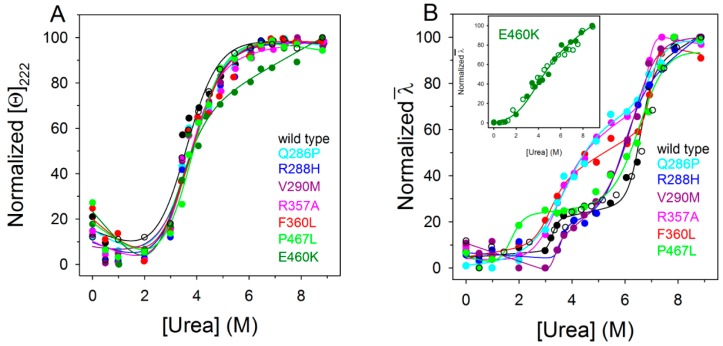
Urea-induced equilibrium unfolding of PPARγ wild type and variants. (**A**) Normalized molar ellipticity at 222 nm ([Θ]_222_) reported after removal of the high-frequency noise and the low-frequency random error by singular value decomposition algorithm (SVD). The continuous lines represent the nonlinear fitting of the normalized molar ellipticities at 222 nm to Equation (3); (**B**) Normalized intensity-averaged emission wavelength (*λ*). The continuous lines represent the three-state fitting of the normalized intensity-averaged emission wavelength data to Equation (5). The inset in (**B**) shows the unfolding of E460K variant fitted according to Equation (3). The reversibility points (empty circles) are shown, for clarity, only for the wild type and for E460K and were not included in the nonlinear regression analysis. All the spectra were recorded at 10 °C, as described in Materials ad Methods.

PPARγ wild type and variants reversibly unfold in urea at 10 °C in 20 mM TrisHCl, pH 8.0, containing 0.2 mM dithiothreitol (DTT) and 0.10 M NaCl. The effect of increasing urea concentrations (0–9 M) on the protein structure was analyzed by far-UV CD and fluorescence spectroscopy. Fluorescence and far-UV CD ellipticity changes during the unfolding transitions were monitored on the same samples. The ellipticity changes at 222 nm induced by urea show a sigmoidal dependence upon denaturant concentration, with an apparent two-state transition without any detectable intermediate (Figure 5A). The unfolding process is fully reversible upon dilution of the denaturant both for the wild type and variants with transition midpoints ranging from 3.82 (Table 1) to 3.00 M urea. The thermodynamic parameters relative to the apparent two-state equilibrium unfolding measured by far-UV CD have been fitted to a two-state model according to Equation (3) and do not indicate any significant difference between the variants and the wild type, except for F360L, which shows a less than 0.5 kcal/mol decrease of ∆*G* of unfolding (Table 1). Notably, the variant E460K also shows a 5.5 degree decrease in thermal stability, displaying a Δ*G*^H^_2_^O^ value closely similar to that of the wild type. The values of *m* generally refer to the amount of protein surface area that becomes exposed to solvent upon unfolding [43]. Interestingly, all the *m* values determined for the PPARγ wild type and its variants, as measured by far-UV CD (Table 1), range between 0.83 and 1.07 kcal/mol/M, values four-fold lower than those predicted for a monomeric protein of 282 amino acid residues unfolded in urea [44,45]. Such low *m* values may be related to multi-state equilibrium unfolding, in line with the results obtained monitoring the unfolding process by intrinsic fluorescence (Figure 5B).

The fluorescence changes induced by increasing urea concentration for PPARγ wild type and all variants (recall that all these proteins lack tryptophan residues) are characterized by an increase in the fluorescence emission intensity and by a broadening of the emission spectra that remain centered at around 308 nm (Figure S2). These spectral changes, analyzed by monitoring the intensity averaged emission wavelength *λ*, show a complex, non-two-state dependence upon increasing urea concentration for the wild type and for the variants Q286P, R288H, V290M, R357A, F360L, and P467L (Figure 5B). The data clearly indicate a three-state unfolding process and the population of a denaturation intermediate above 3.50 M (Figures 5B and S2), about the same urea concentration of the apparent denaturation midpoints observed by monitoring the ellipticity changes, with the exception of P467L, whose fluorescence intermediate becomes apparent above 2.0 M urea (Figures 5B and S2). The three-state transitions monitored by fluorescence, are not coincident with the two-state transitions monitored by far-UV CD and were fitted to a three-state unfolding process according to Equation (5), yielding the thermodynamic parameters reported in Table 2.

For the first transition, which represents the unfolding of the native to the intermediate state, Δ*G*^H^_2_^O^_I–N_ values of R288H and V290M are similar to those of the wild type, whereas those of P467L, F360L, R357A, and Q286P are significantly lower, suggesting a destabilization of the native state for these variants (Table 2). For the second transition, which represents the unfolding of the intermediate to the denatured state, the Δ*G*^H^_2_^O^_U–I_ values of R357A and F360L are higher than those of the wild type, suggesting that the intermediate state of the two variants is more stable. In the case of Q286P, R288H, V290M, and P467L, the Δ*G*^H^_2_^O^_U–I_ values are about half those of the wild type and suggest a less stable intermediate (Table 2). The differences in the values of Δ*G*^H^_2_^O^, from the native to the intermediate state and from the intermediate to the unfolded state, are mainly due to differences in *m* values, i.e., in the solvent exposed surface area upon unfolding. Notably, for the variants Q286P, R357A, F360L, and P467L, the *m* value from the native to the intermediate state is significantly lower than that of the wild type; in the transition from the intermediate to the unfolded state is observed a decrease of *m* value of Q286P, R288H, V290M, and P467L and an increase of *m* value of R357A and F360L. Taken together, these results indicate for all the variants a total Δ*G*^H^_2_^O^ value, relative to the unfolding from the native to the denatured state, lower than that of the wild type and suggest a destabilization of the native state for Q286P, R357A, F360L, and P467L and a stabilization of the intermediate state of R357A and F360L. In the case of E460K, which shows a shallow unfolding transition, the changes of intensity averaged emission wavelength *λ* at increasing urea concentration were fitted to a two-state unfolding process, according to Equation (3), yielding Δ*G*^H^_2_^O^, *m* and [Urea]_0.5_ values of 1.81 ± 0.3 kcal/mol, 0.54 ± 0.08 kcal/mol/M and 3.37 M, respectively (Figure 5B, inset).

### 2.5. Molecular Dynamics

MD simulations are invaluable in interpreting experimental data since they allow us to follow at the atom level the changes occurring in each of the mutant proteins. The basic data concerning the 10 (nine mutants plus the wild type) simulated systems are reported in Table 3. After 40 ns of equilibration, we followed the simulated systems for another 110 ns in the NVT *ensemble*. From the collected configurations we computed the backbone root mean square deviations (r.m.s.d.) of each PPARγ variant with respect to the wild type starting structure (PDB ID: 1PRG) as a function of the simulation time. From this analysis we conclude that only the R357A and R397C r.m.s.d. are significantly different from the wild type r.m.s.d., meaning that these two variants are structurally the most distant ones from the PPARγ wild-type crystalline state. Moreover, a calculation of the gyration radius shows that R357A is the most compact system as it has the smallest gyration radius among all the mutants (Table 3), significantly smaller than that of the wild type. The other two interesting parameters that we found useful to monitor along the MD trajectory are the distances between H3 and helix 12 (H12) and between H12 and subportion 280–287 of PPARγ. In Table 3 (the last two columns) we report the mean value and the standard deviation of these two distances computed along the last 110 ns of the trajectory. In the Q286P variant, the H3–H12 distance is considerably smaller than in the wild type. In R288H and R357A we notice a large standard deviation due to the fact that H3–H12 distance oscillates. The F360L variant is the only one for which both the H3–H12 distance and the distance between H12 and the PPARγ 280–287 subportion are significantly larger (beyond errors) than in the wild type. This can be interpreted by saying that in the case of the F360L variant the strength of the inter-helical interactions is considerably reduced.

H3 appears to undergo secondary structural changes in four of the analyzed mutants, namely Q286P, R357A, F360L, and R397C. In Q286P, H3 assumes a 3-helix and turn secondary structure in the 277–287 region. In R357A it takes a coil structure in the 286–292 segment. In F360L it becomes turn and 3-helix in the 280–288 region, while in R397C its structure changes in a long segment 287–302 assuming a turn and a 3-helix secondary structure. In the wild type and in all the other variants, H3 stably remains in an α-helix structure. Compared to H3, the secondary structure of H12 is generally less stable. The reason is that H12 is at the C-terminal, hence it is located in a rather mobile region. H12 completely loses its α-helix secondary structure in favor of a turn structure only in R357A, F360L, and P467L.

**Figure 6:**
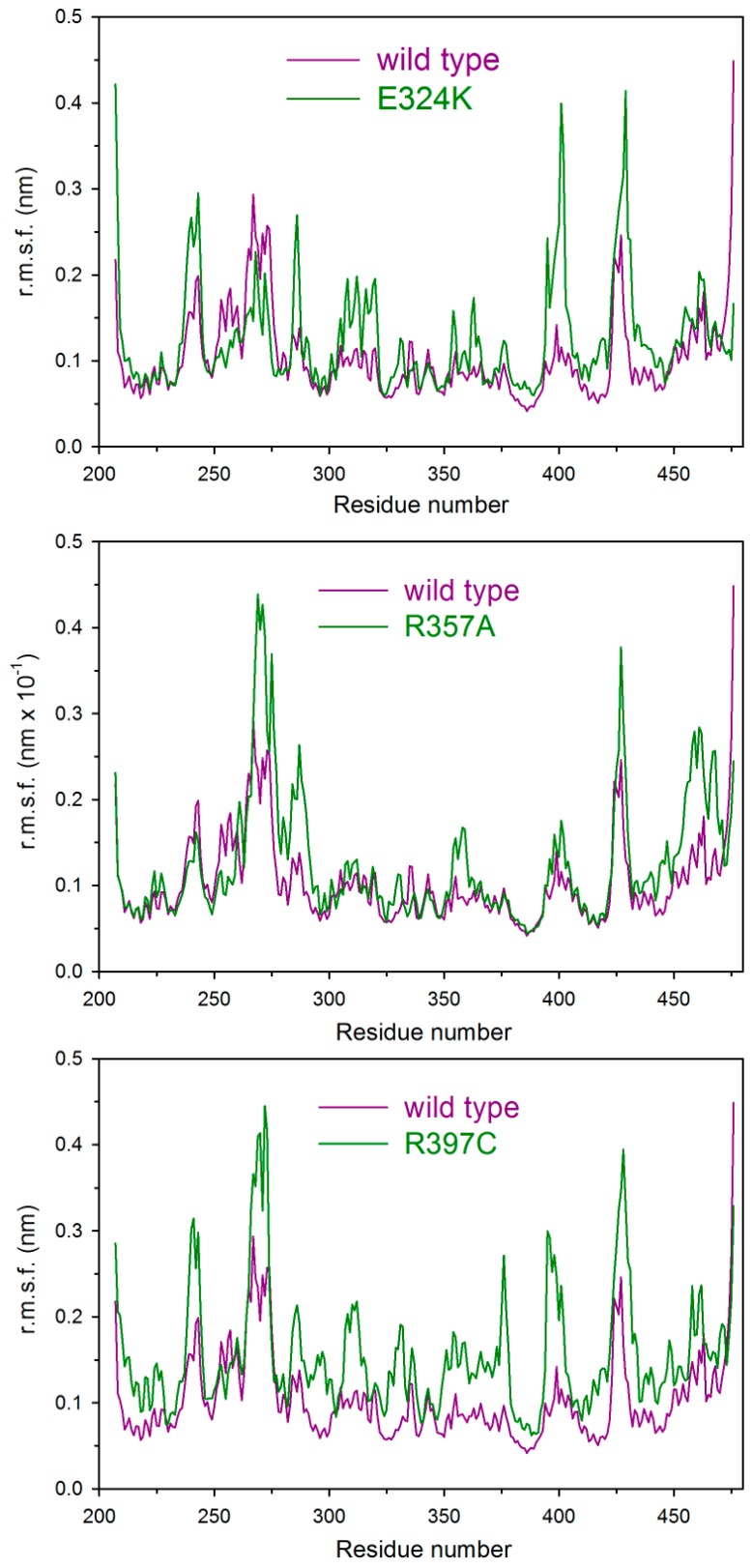
Mobility per residue of PPARγ wild type and E324K, R357A, and R397C variants. Cα root mean square fluctuations (r.m.s.f.) per residue for E324K, R357A, and R397C variants compared with the wild type r.m.s.f. On the *x*-axis is the residue number and on the *y*-axis is the mobility in nm.

The analysis of the Cα root mean square fluctuations (r.m.s.f.) per residue shows that the point mutations significantly alter the PPARγ mobility. In Figure 6 we show the r.m.s.f. of the three variants, E324K, R357A, and R397C, whose mobility is definitely higher than that of the wild-type protein. One notices that R397C is the PPARγ variant with the highest mobility and the largest number of involved residues.

By following the history of specific residues along the simulated trajectories, we can monitor the stability of some structurally important salt bridges. We have examined the history of the E259–R280, E324–R397, and E460–R357 salt-bridges along the MD history. The E259–R280 salt bridge is absent in the wild type, and is present only in the E324K, R357A, and R397C variants. The salt bridges E324–R397 and E460–R357 are always present and stable except in the variants where one of the amino acids involved in the salt bridge is mutated (E324K and R397C for the first salt bridge; E460K and R357A for the second). It is worth noting that the R357–E460 distance is more stable and smaller in the Q286P variant (Figure S3A) than in all the other variants and in the wild type.

The E276–R357 salt bridge is lost in the R357A variant (because of the point mutation), while it is quite stable in all the other cases, except the wild type and the E460K variant (Figure S3B). In Figure S3B we report the E276–R357 distance in the case of the E324K variant as an example of stability. Finally, we monitored the distance between the R397 and R443 residues. We found that these two residues are always rather near except in the E324K and R397C variants. A possible explanation of such behavior is that the absence of the E324–R397 salt bridge in these two variants causes the residue R397 to move away from R443. The largest oscillations of R397–R443 distance are found in the Q286P variant even if the two residues remain closer than in E324K and R397C. In Figure S3C we report the time evolution of the R397–R443 distance in the case of the Q286P variant together with that of the wild type and the F360L variant. The latter is shown just to compare with a case where oscillations are small.

### 2.6. Transcription Activity

The transcription activity of F360L, R357A, P467L, and Q286P PPARγ variants was evaluated in comparison with wild-type PPARγ LBD in the presence of the full agonist rosiglitazone and LT175, a partial agonist that binds to a different region of PPARγ. For this purpose, GAL4–PPAR chimeric receptors were expressed in transiently transfected HepG2 cells according to a previously reported procedure [46]. As previously reported, the efficacy of both ligands remained basically unchanged towards F360L compared to the wild type, while the potency was significantly reduced [25]. A remarkable lowering in both efficacy and potency was shown for R357A and P467L (Figure S4 and Table S1). In particular, rosiglitazone displayed a remarkable lowering of potency; its EC_50_ value, in fact, was 7-fold higher against R357A and 18-fold higher against P467L compared to the wild type, whereas for LT175 this value turned out to be only about twice as high (Table S1). Singular behavior has been observed for the mutant Q286P, which was completely inactive and insensitive to both rosiglitazone and LT175 (Figure S5).

## 3. Discussion

In the post-genomic era, how human genetic and somatic variations are associated with diseases and how mechanisms form the basis of the relationship between genotype and phenotype are still open questions. Genetic polymorphism is responsible for phenotypic differences among humans and individual susceptibility to genetic disease and therapeutic responses. nsSNPs are of particular interest since the single-nucleotide variations occurring in the DNA coding region lead to a polypeptide with a change in the amino acid sequence that may affect the structure and/or function of the protein. The structural analysis of nsSNP protein variants may help in understanding the molecular basis of diseases and, since individuals carrying variants may respond differently to drugs, it may provide information for personalized drugs tailored to the individual variant. For complex diseases such as cancer and diabetes, SNPs may not function individually; rather, they work in coordination with other SNPs to manifest a disease condition. However, nsSNP variants may be utilized as efficient structural and functional markers of association with complex diseases.

Experimental [25,27,28,40] and computational [47,48,49,50] studies on several proteins related the effect of nsSNPs to the alteration of protein stability, protein–protein interactions, and protein functions. The interest in studying the effects of nsSNPs on structural stability and dynamic properties of PPARγ derives from the involvement of this nuclear receptor in a variety of biological processes such as adipocyte differentiation and insulin sensitization, as well as cellular differentiation and development and carcinogenesis [14]. Notably, PPARγ functions have been linked to several pathologies, ranging from metabolic disorders to cardiovascular disease, chronic inflammation, neurodegenerative disorders, and cancer [51,52]. PPARs ligands and other agents influencing PPAR signaling pathways have been shown to display chemopreventive potential by mediating tumor suppressive activities in a variety of human cancers and could represent novel targets to inhibit carcinogenesis and prevent tumor progression [53]. In addition, PPARγ agonists have recently been reported to lower the incidence of a number of neurological disorders [54]. All these functions are accomplished by binding PPARγ LBD to different ligands, which leads to conformational changes that promote the interaction with coactivator proteins in the nucleus [55].

PPARγ, a nuclear receptor that belongs to the ligand-dependent transcription factors, consists of a central DNA binding domain and a carboxy-terminal domain involved in ligand binding, dimerization, and transactivation. PPARγ adopts an active conformation that promotes transcription upon heterodimerization with RXR in the presence of a ligand. The ligand binding site is buried within the core of the LBD, which is folded into three layers of α-helices (Figures 2 and S1) [2].

Missense mutations in PPARγ LBD caused by nsSNPs may induce profound phenotypic changes in affected individuals, contributing to the risk of onset of various pathological states, like dyslipidemia, type 2 diabetes [16], and cancer [8,13,17,18,19,20,21,22,23]. The molecular mechanism that leads to the loss and/or alteration of PPARγ functions in nsSNP variants is not clear [23,24]. In this study we investigate the effect of the mutations on PPARγ LBD; to our knowledge, this is the first report that analyzes, in comparison with the wild type, nine PPARγ non-synonymous polymorphic variants of the LBD in terms of their spectroscopic properties in solution, thermodynamic and thermal stability, and molecular dynamics. The selection of the variants was focused on those mutations located in putatively critical positions, such as Q286P, R288H, V290M, E324K, E460K, and P467L, in close proximity to the residues involved in ligand binding (Figures 1 and S1). We also considered those non-conservative amino acid substitutions leading to alteration of the polarity of the residue, such as E324K, E460K, R357A, and R397C, or in the secondary structure propensity, as in the case of Q286P. All PPARγ variants were obtained as recombinant soluble proteins, with the exception of E324K and R397C, which could not be expressed in the soluble fraction even when different induction conditions were used. Interestingly, E324 and R397, located on H5 and on a loop, respectively, are both involved in one of the two salt bridges that may contribute to PPARγ stabilization (Figure 2C,D). The importance of the two salt bridges (Figure 2C,D) is also evident from the consequence of the mutation of the negatively charged E460 into a positively charged lysine at the end of H12, which breaks the salt bridge network formed by R357 and E276, both located on a loop. The importance of this salt bridge network, and of R357in particular, has already been described by the effect of its mutation into alanine on the global stabilization of the entire LBD [25]. As a matter of fact, E460K shows the lowest melting temperature, five degrees lower than that of the wild type, and a poorly cooperative urea-induced unfolding transition monitored by fluorescence changes, characterized by very low values of thermodynamic parameters (Figure 5B inset). Notably, the thermodynamic parameters, measured by monitoring the secondary structure changes by far-UV circular dichroism in the apparent two-state urea-induced unfolding transitions, are similar for all variants and only slightly different with respect to the wild type, with the exception of F360L, which shows ∆*G*^H^_2_^O^, *m*, and T_m_ values lower than those of the wild type (Table 1). These results suggest a similar overall secondary structure folding for all variants with respect to the wild type. On the other hand, the tertiary structure changes monitored by fluorescence reveal a complex non two-state process and significant differences among the natural variants. The analysis of the thermodynamic parameters obtained by fitting the fluorescence changes to a three-state unfolding reveals a decreased stability of the native state for all variants except for R288H and V290M. Interestingly, the variants P467L and Q286P show a destabilization of both the native and the intermediate state and are inactive. Both amino acid substitutions involve a proline residue and, in the case of Q286P, a residue located in the middle of H3; its functional relevance has been previously addressed in [56]. Tertiary structural variations between the wild type and variants are indicated by comparison of their near-UV CD spectra; in particular, amino acid substitutions in the variants F360L, P467L, and Q286P lead to changes in the overall protein tertiary arrangements, and minor tertiary changes are observed for all the other variants. Notably, all variants show a slight decrease in inter-helical interactions, as suggested by the decrease of 222/208 ellipticity ratio, more significant for F360L. These results, taken together, suggest a possible increase in the flexibility of the variants with respect to the wild type, as confirmed by molecular dynamics simulations. The most flexible variants (Figure 6) are E324K, R357A, and R397C, precisely the ones where the mutation affects a residue involved in one of the salt bridges that are supposed to contribute to the PPARγ LBD wild-type stabilization. Moreover, molecular dynamics simulations are able to confirm the presence of small changes in the secondary structure of all the variants compared to the wild type and a more significant decrease of inter-helical interactions for the F360L variant (the last two columns of Table 3). The importance of inter-helical interactions and the correct reciprocal positioning of H3 and H12 has been previously reported as a crucial point for PPARγ function [57].

## 4. Materials and Methods

### 4.1. Plasmids and Site-Directed Mutagenesis

The LBD of PPARγ wild type (gene ID 5468, amino acids 174–477, expected molecular mass 34.5 kDa) and mutants were cloned in pET-28 plasmid for *Escherichia coli* expression as previously described [58]. The plasmid harboring the PPARγ wild-type gene was used to obtain mutant enzymes. The QuikChange Site-Directed Mutagenesis Kit (Stratagene, San Diego, CA, USA) was used to introduce the point mutations into the bacterial expression vector and into the vector expressing the chimeric receptor containing the yeast Gal4 DNA-binding domain fused to the wild-type PPARγ LBD used for the transcription activity assay [59]. The mutagenic synthetic oligonucleotides are shown in Table S2. Sequence analysis was performed to confirm the presence of the desired mutations and the absence of additional mutations.

### 4.2. Protein Preparation

PPARγ isoform 1 (UniProt ID P37231-2) wild type and mutants (Table S3) were expressed as N-terminally His-tagged proteins using a pET-28 vector and then purified as follows. *E. coli* KRX cells were transformed with the selected plasmid and were grown on an LB medium with 30 mg/mL kanamycin at 37 °C to an OD of 0.6. The culture was then induced with 5.0 mM rhamnose and further incubated at 18 °C for 20 h with vigorous shaking. Cells were collected by centrifugation and resuspended as a 20 mL culture in buffer A (20 mM Tris, 150 mM NaCl, 10% glycerol, 1 mM tris(2-carboxyethyl)phosphine–HCl (TCEP) pH 8.0) in the presence of protease inhibitors (Complete Mini EDTA-free; Roche Applied Science, Monza, Italy). The cells were sonicated and the soluble fraction was isolated by centrifugation (35,000× *g* for 45 min). The supernatant was applied to a Ni^2+^–nitrilotriacetic acid column (GE Healthcare) and elution was performed with 0.25 M imidazole in buffer A. The pure fractions were concentrated to 2 mL using Millipore (Milano, Italy), concentrators and loaded onto a Superdex, 75 10/300, GE Healthcare (Milano, Italy), gel-filtration column on an ÄKTA FPLC system previously equilibrated with 50 mM Tris–HCl, 0.25 M NaCl, 2 mM DTT pH 8.0 at a flow rate of 1.0 mL/min. The eluates were collected and SDS–PAGE was used to test the purity of the protein. The proteins were identified by mass-spectrometric analysis. SDS–PAGE bands were cut from the gel and processed via tryptic proteolysis. The peptide mixtures were analyzed by a MALDI-ToF, AutoFlex II (Bruker Daltonics, Bremen, Germany) mass spectrometry instrument. Data were manually analyzed by a FlexAnalysis program (Bruker Daltonics) that revealed the expected site mutations according to a theoretical mass list of tryptic PPARγ peptides. The protein was then cleaved with thrombin protease (GE Healthcare (Milano, Italy); 10 U/mg) at room temperature for 2 h. The digested mixture was reloaded onto an Ni^2+^–nitrilotriacetic acid column to remove the His tag and the undigested protein. The flowthrough was loaded onto a Q-Sepharose HP column (GE Healthcare) and eluted with a 0–500 mM gradient of NaCl in buffer B (20 mM Tris, 10% glycerol, 1 mM TCEP pH 8.0) with a BioLogic DuoFlow FPLC system (Bio-Rad Laboratories, Milano, Italy). Finally, the protein was purified by gel-filtration chromatography on a HiLoad Superdex 75 column (GE Healthcare) and eluted with buffer C (20 mM Tris, 1 mM TCEP, 0.5 mM EDTA pH 8.0). Protein quantification was determined according to OD_280_ measurement using the respective molar extinction coefficients ε of each protein, calculated according to [60].

### 4.3. Cell Culture and Transfections

Human hepatocellular liver carcinoma cell line HepG2 (Interlab Cell Line Collection, Genoa, Italy) was cultured in Minimum Essential Medium (MEM) containing 10% heat-inactivated fetal bovine serum, 100 U/mL of penicillin G, and 100 μg/mL of streptomycin sulfate at 37 °C in a humidified atmosphere of 5% CO_2_ (250 ng). For transactivation assays, 1 × 10^5^ cells per well were seeded in a 96-well plate and transfected after 24 h with K2 Transfection System (Biontex Laboratories GmbH, Munchen, Germany) according to the manufacturer’s protocol using 0.20 µg/well of DNA. Cells were transfected with expression plasmids encoding the fusion protein Gal4–PPARγ–LBD (wild type, P467L, or Q286P mutant), pGal5TKpGL3, and pCMVβgal to normalize the transfection efficacy. Twenty-four hours after transfection, the medium was replaced with a fresh medium supplemented with rosiglitazone (ranging from 2 nM to 10 µM), LT175 (ranging from 100 nM to 10 µM) or DMSO 0.1%. After a further 24 h of incubation, cells were lysed and the luciferase activity in cell extracts was determined by a luminometer (VICTOR^3^ V Multilabel Plate Reader, Perkin-Elmer, Monza, Italy) and normalized for β-galactosidase activity. Fold induction activity was calculated and plotted using GraphPad Prism 5.04 software (La Jolla, CA, USA). All transfection experiments were repeated at least twice with similar results. The results were expressed as mean ± SEM.

### 4.4. Spectroscopic Measurements

Intrinsic fluorescence emission spectra were recorded from 290 to 440 nm (274 nm excitation wavelength, 1 nm sampling interval), at 0.1 mg/mL protein concentration (3.25 × 10^−2^ AU at 280 nm) in 20 mM Tris–HCl pH 8.0 containing 0.1 M NaCl and 0.2 mM DTT with a LS50B spectrofluorimeter (Perkin-Elmer) using a 1.0 cm path length quartz cuvette. Far-UV (190–250 nm) CD spectra were monitored at a protein concentration of 200 μg/mL (6.50 × 10^−2^ AU at 280 nm) in 50 mM Tris-Cl pH 8.0, 0.2 mM DTT, 0.2 M NaCl, using a 0.1 cm path length quartz cuvette. Near-UV (250–320 nm) CD spectra were monitored at a protein concentration of 4.6 mg/mL (1.49 AU at 280 nm) in 50 mM Tris-Cl pH 8.0, 2.0 mM DTT, 0.2 M NaCl, in a 1.0 cm path length quartz cuvette. CD measurements were performed in a JASCO-815 spectropolarimeter (Jasco, Easton, MD, USA) and the results were expressed as the mean residue ellipticity ([Θ]), assuming a mean residue molecular mass of 110 per amino acid residue. All spectroscopic measurements were carried out at 10 °C.

### 4.5. Urea-Induced Equilibrium Unfolding

For equilibrium transition studies, PPARγ wild type and variants (final concentration ranging over 100–200 μg/mL) were incubated at 10 °C at increasing concentrations of urea (0−9 M) in 20 mM Tris/HCl, pH 8.0, in the presence of 0.2 M NaCl and 200 μM DTT. After 10 min, equilibrium was reached and intrinsic fluorescence emission and far-UV CD spectra (0.1-cm cuvette) were recorded in parallel at 10 °C. To test the reversibility of the unfolding, PPARγ wild type and variants were unfolded at 10 °C in 8.0 M urea at protein concentration ranging over 1.0–2.0 mg/mL in 20 mM Tris/HCl, pH 8.0, in the presence of 2 mM DTT and 0.2 M NaCl. After 10 min, refolding was started by 10-fold dilution of the unfolding mixture at 10 °C into solutions of the same buffer used for unfolding containing decreasing urea concentrations. The final protein concentration ranged over 100–200 μg/mL. After 24 h, intrinsic fluorescence emission and far-UV CD spectra were recorded at 10 °C. All denaturation experiments were performed in triplicate.

### 4.6. Thermal Denaturation Experiments

PPARγ wild type and variants (protein concentration ranging over 0.10–0.20 mg/mL) were heated from 20 to 75 °C in a 0.1 cm quartz cuvette with a heating rate of 1 degree × min^−1^ controlled by a Jasco programmable Peltier element (Jasco, Easton, MD, USA). The dichroic activity at 222 nm and the photomultiplier voltage (PMTV) were continuously monitored in parallel every 0.5 °C [61]. All the thermal scans were corrected for the solvent contribution at the different temperatures. Melting temperature (T_m_) values were calculated by taking the first derivative of the ellipticity at 222 nm with respect to temperature. All denaturation experiments were performed in triplicate.

### 4.7. Data Analysis

Far-UV CD spectra recorded as a function of urea concentration were analyzed by a singular value decomposition algorithm (SVD) using the software MATLAB (Math-Works, South Natick, MA, USA) to remove the high-frequency noise and the low-frequency random errors and determine the number of independent components in any given set of spectra, as described in [40].

The changes in intrinsic fluorescence emission spectra at increasing urea concentrations were quantified as the intensity-averaged emission wavelength, *λ*, [62] calculated according to

(1) $$\bar{\lambda }=\sum (I_{i}\lambda_{i})/\sum (I_{i})$$

where *λ*_i_ and *I*_i_ are the emission wavelength and its corresponding fluorescence intensity at that wavelength, respectively. This quantity is an integral measurement, negligibly influenced by the noise, which reflects changes in the shape and position of the emission spectrum.

Urea-induced equilibrium unfolding transitions monitored by far-UV CD ellipticity and intrinsic fluorescence emission changes were analyzed by fitting baseline and transition region data to a two-state linear extrapolation model [63] according to

(2) $$△G_{unfolding}=△G^{H_{2}O}+m\left[\mathrm{Urea}\right]=-RT\ln K_{unfolding}$$

where ∆*G*_unfolding_ is the free energy change for unfolding for a given denaturant concentration, ∆*G*^H^_2_^O^ is the free energy change for unfolding in the absence of denaturant, *m* is a slope term that quantifies the change in ∆*G*_unfolding_ per unit concentration of denaturant, *R* is the gas constant, *T* is the temperature and *K*_unfolding_ is the equilibrium constant for unfolding. The model expresses the signal as a function of denaturant concentration:

(3) $$y_{i}=\frac{y_{N}+s_{N}{\left[X\right]}_{i}+(y_{U}+s_{U}{\left[X\right]}_{i})\times \exp\left[(-△G^{H_{2}O}-m{\left[X\right]}_{i}/RT\right]}{1+\exp\left[\frac{(-△G^{H_{2}O}-m{\left[X\right]}_{i}}{RT}\right]}$$

where *y*_i_ is the observed signal; *y*_U_ and *y*_N_ are the baseline intercepts for unfolded and native protein, respectively; *s*_U_ and *s*_N_ are the baseline slopes for the unfolded and native protein, respectively; [*X*]_i_ is the denaturant concentration after the *i*th addition; ∆*G*^H^_2_^O^ is the extrapolated free energy of unfolding in the absence of denaturant, and *m* is the slope of a ∆*G*_unfolding_ versus [*X*] plot. The denaturant concentration at the midpoint of the transition, [Urea]_0.5_, according to Equation (2), is calculated as:

(4) $${\left[\mathrm{Urea}\right]}_{0.5}=△G^{H_{2}O}/m$$

The denaturation curve obtained by plotting the fluorescence changes of the PPARγ wild type and variants induced by increasing urea concentrations was fitted to the following equation assuming a three-state model:

(5) $$F=\frac{F_{N}+\exp\left(m_{I-N}\frac{\left[\mathrm{urea}\right]-D50_{I-N}}{RT}\right)\times \left(F_{I}+F_{U}\exp\left(m_{U-I}\frac{\left[\mathrm{urea}\right]-D50_{U-I}}{RT}\right)\right)}{1+\exp\left(m_{I-N}\frac{\left[\mathrm{urea}\right]-D50_{I-N}}{RT}\right)\times \left(1+\exp\left(m_{I-N}\frac{\left[\mathrm{urea}\right]-D50_{I-N}}{RT}\right)\right)}$$

where *F* is *λ*, calculated according to Equation (1); *m* is a constant that is proportional to the increase in solvent-accessible surface area between the two states involved in the transition; *D*50_I–N_ and *m*_I–N_ are the midpoint and *m* value for the transition between N and I, respectively; and *D*50_U–I_ and *m*_U–I_ are the midpoint and *m* value for the transition between I and U, respectively [64]. The λ of the intermediate state (I), *F*_I_, is constant, whereas that of the folded state (N) and of the unfolded state (U), *F*_N_ and *F*_U_, respectively, has a linear dependence on denaturant concentration:

(6) $$F_{N}=a_{N}+b_{N}\left[\mathrm{urea}\right]$$

(7) $$F_{U}=a_{U}+b_{U}\left[\mathrm{urea}\right]$$

where *a*_N_ and *a*_U_ are the baseline intercepts for N and U, and *b*_N_ and *b*_U_ are the baseline slopes for N and U, respectively. All unfolding transition data were fitted using Graphpad Prism 5.04 (La Jolla, CA, USA).

### 4.8. Molecular Dynamics Simulations

Molecular Dynamics (MD) simulations were performed with the GROMACS package [65,66,67,68]. The initial coordinates of the wild-type protein were taken from the crystal structure of the PPARγ receptor [2] (PDB ID: 1PRG). The coordinates of the nine variants were adapted from the wild-type coordinates by performing a point mutation. Each system was placed in a dodecahedral box of sufficiently large dimensions such that nearby images lay more than 10 Å away. The box was filled with water molecules and an appropriate number of counter-ions to make the whole system neutral. As in [25], an OPLS force field [69] was used to simulate PPARγ and all its variants.

The equilibration strategy adopted for the nine systems is quite standard and is explained in detail in [70,71]. The temperature was held fixed at 300 K using the v-rescale thermostat [72] with a coupling time of 0.1 ps. The single point charge (SPC) model was employed for water molecules. Periodic boundary conditions were used throughout the simulation. Coulomb interactions have been dealt with by a standard Particle Mesh Ewald algorithm [73]. A time step of 2 fs was used. A non-bond pair list cutoff of 1.0 nm was used. The list was updated every 10 steps.

Each one of the 10 systems was simulated for 120 ns in the NVT ensemble. The analysis of the numerical data obtained in the simulation was carried out by GROMACS and VMD [74] tools according to needs.

## 5. Conclusions

In conclusion, the nine nsSNP PPARγ variants associated with metabolic disorders and /or cancer show alterations in the dynamics and tertiary contacts that impair the correct reciprocal positioning of H3 and H12, crucially important for PPARγ functioning. These alterations may lead to changes in the interactions with ligands and influence the multiple biological functions of this nuclear receptor.

## Figures and Tables

**Table 1:** Melting temperatures and thermodynamic parameters for urea-induced unfolding equilibrium of PPARγ wild type and mutants measured by far-UV CD spectroscopy.

PPARγ	T_m_ (°C)	∆*G*^H^_2_^O^ (kcal/mol)	*m* (kcal/mol/M)	[Urea]_0.5_ (M)
Wild type	49.5	3.37 ± 0.15	0.95 ± 0.05	3.16
Q286P	48.0	3.07 ± 0.15	0.84 ± 0.05	3.65
R288H	50.0	3.43 ± 0.12	0.94 ± 0.04	3.65
V290M	49.5	3.40 ± 0.10	0.89 ± 0.03	3.82
R357A	48.0	3.56 ± 0.10	1.00 ± 0.03	3.56
F360L	46.5	2.97 ± 0.10	0.83 ± 0.03	3.58
P467L	50.0	3.48 ± 0.11	0.93 ± 0.04	3.74
E460K	44.0	3.20 ± 0.16	1.07 ± 0.06	3.00

The temperature-induced changes were followed by monitoring the ellipticity at 222 nm. The T_m_ values were calculated by taking the first derivative of the ellipticity at 222 nm with respect to temperature. Urea-induced unfolding equilibrium data were measured at 10 °C in 20 mM Tris/HCl, pH 8.0, containing 0.2 M NaCl and 200 μM DTT by monitoring ellipticity at 222 nm [Θ_222_]. ∆*G*^H^_2_^O^ and *m* values were obtained from Equation (3); [Urea]_0.5_ was calculated from Equation (4). Data are reported as the mean ± SE of the fit.

**Table 2:** Thermodynamic parameters for urea-induced unfolding equilibrium of PPARγ wild type and mutants measured by fluorescence spectroscopy.

PPARγ	*m*_I–N_ (kcal/mol/M)	*D*50_I–N_ (M)	Δ*G*^H^_2_^O^_I–N_ (kcal/mol)	*m*_U__–__I_ (kcal/mol/M)	*D*50_U–I_ (M)	Δ*G*^H^_2_^O^_U–I_ (kcal/mol)
Wild type	5.30 ± 0.64	3.26 ± 0.09	17.27	2.47 ± 0.32	6.56 ± 0.04	16.20
Q286P	0.85 ± 0.10	3.73 ± 0.07	3.17	1.53 ± 0.18	7.00 ± 0.11	10.76
R288H	5.24 ± 0.52	3.57 ± 0.09	18.71	1.42 ± 0.17	5.89 ± 0.07	8.36
V290M	4.96 ± 0.64	3.32 ± 0.18	16.47	1.29 ± 0.14	6.41 ± 0.05	8.27
R357A	1.21 ± 0.16	3.31 ± 0.06	4.01	4.71 ± 0.50	6.88 ± 0.04	32.40
F360L	1.87 ± 0.22	3.09 ± 0.14	5.79	3.71 ± 0.48	6.86 ± 0.06	25.45
P467L	2.48 ± 0.24	1.66 ± 0.24	4.12	1.13 ± 0.16	6.58 ± 0.08	7.43

Urea-induced unfolding equilibrium data were obtained at 10 °C in 20 mM Tris/HCl, pH 8.0, containing 0.2 M NaCl and 200 μM DTT by measuring the fluorescence intensity averaged emission wavelength *λ*. The free energy of unfolding from the native state to the intermediate (Δ*G*^H^_2_^O^_I_**_–_**_N_) and from the intermediate to the unfolded state (Δ*G*^H^_2_^O^_U_**_–_**_I_) were calculated from Equation (5). *D*50_I_**_–_**_N_ and *m*_I_**_–_**_N_ which are the midpoint and *m* value for the transition between native and intermediate state, respectively, and *D*50_U_**_–_**_I_ and *m*_U_**_–_**_I_ are the midpoint and *m* value for the transition between the intermediate and the unfolded state, respectively, were calculated from Equation (5). Data are reported ± SE of the fit.

**Table 3:** MD results for PPARγ wild type and the nine mutants.

System	Backbone r.m.s.d. (nm)	Gyration Radius (nm)	H3–H12 Distance (nm)	H12 Subportion (280–287) Distance (nm)
wild type	0.28 (0.02)	1.96 (0.01)	1.14 (0.05)	1.45 (0.05)
Q286P	0.26 (0.01)	1.96 (0.01)	1.00 (0.05)	1.42 (0.06)
R288H	0.26 (0.02)	1.95 (0.01)	1.2 (0.1)	1.46 (0.06)
V290M	0.28 (0.02)	1.95 (0.01)	1.15 (0.05)	1.40 (0.03)
E324K	0.28 (0.02)	1.94 (0.01)	1.05 (0.04)	1.39 (0.04)
R357A	0.39 (0.02)	1.92 (0.01)	1.3 (0.1)	1.5 (0.1)
F360L	0.28 (0.02)	1.98 (0.01)	1.44 (0.06)	1.60 (0.06)
R397C	0.38 (0.03)	1.98 (0.01)	1.26 (0.05)	1.45 (0.08
E460K	0.29 (0.02)	1.97 (0.01)	1.10 (0.06)	1.42 (0.05)
P467L	0.28 (0.02)	1.95 (0.02)	1.13 (0.06)	1.41 (0.05)

The r.m.s.d., the gyration radius, the H3–H12, and H12 subportion (280–287) distance mean values and (standard deviations) are computed on the last 110 ns of simulation.

## Supplementary Materials

Supplementary materials can be found at www.mdpi.com/1422-0067/18/2/361/s1.

## Abbreviations

DTT	Dithiothreitol
LBD	Ligand-Binding Domain
MD	Molecular Dynamics
nsSNPs	Non-synonymous single-nucleotide polymorphisms
PPARγ	Peroxisome Proliferator-Activated Receptor γ
