# Supplementary material for: Single-Nucleotide Polymorphism of PPARγ, a Protein at the Crossroads of Physiological and Pathological Processes
Source: Int J Mol Sci. 2017 Feb 10;18(2):361. doi: 10.3390/ijms18020361 (PMC5343896; doi:10.3390/ijms18020361)
Supplement: Supplementary file 1 [file ijms-18-00361-s001.pdf]

Supplementary Materials: Single Nucleotide Polymorphism of PPARγ, A Protein at the Crossroads of Physiological and Pathological Processes

Maria Petrosino, Laura Lori, Alessandra Pasquo, Clorinda Lori, Valerio Consalvi, Velia Minicozzi, Silvia Morante, Antonio Laghezza, Alessandra Giorgi, Davide Capelli and Roberta Chiaraluce

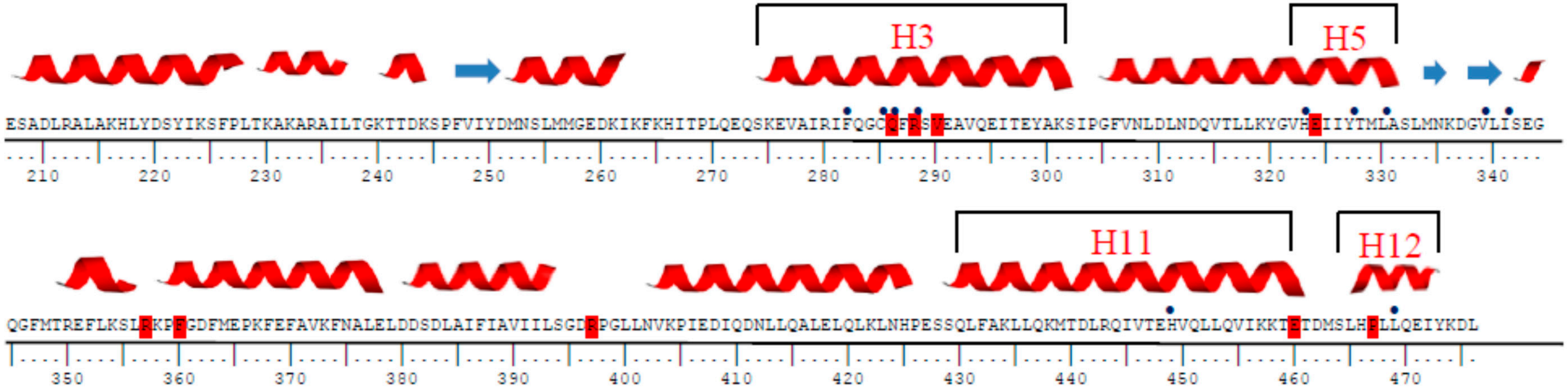

Figure S1. Amino acid sequence of PPARγ LBD. Secondary structure elements are shown at the top of the amino acid sequence. Mutated residues are highlighted in red. Blue dots indicate the residues involved in ligand binding (1PRG.pdb). Helices are numbered according to Nolte et al. [1].

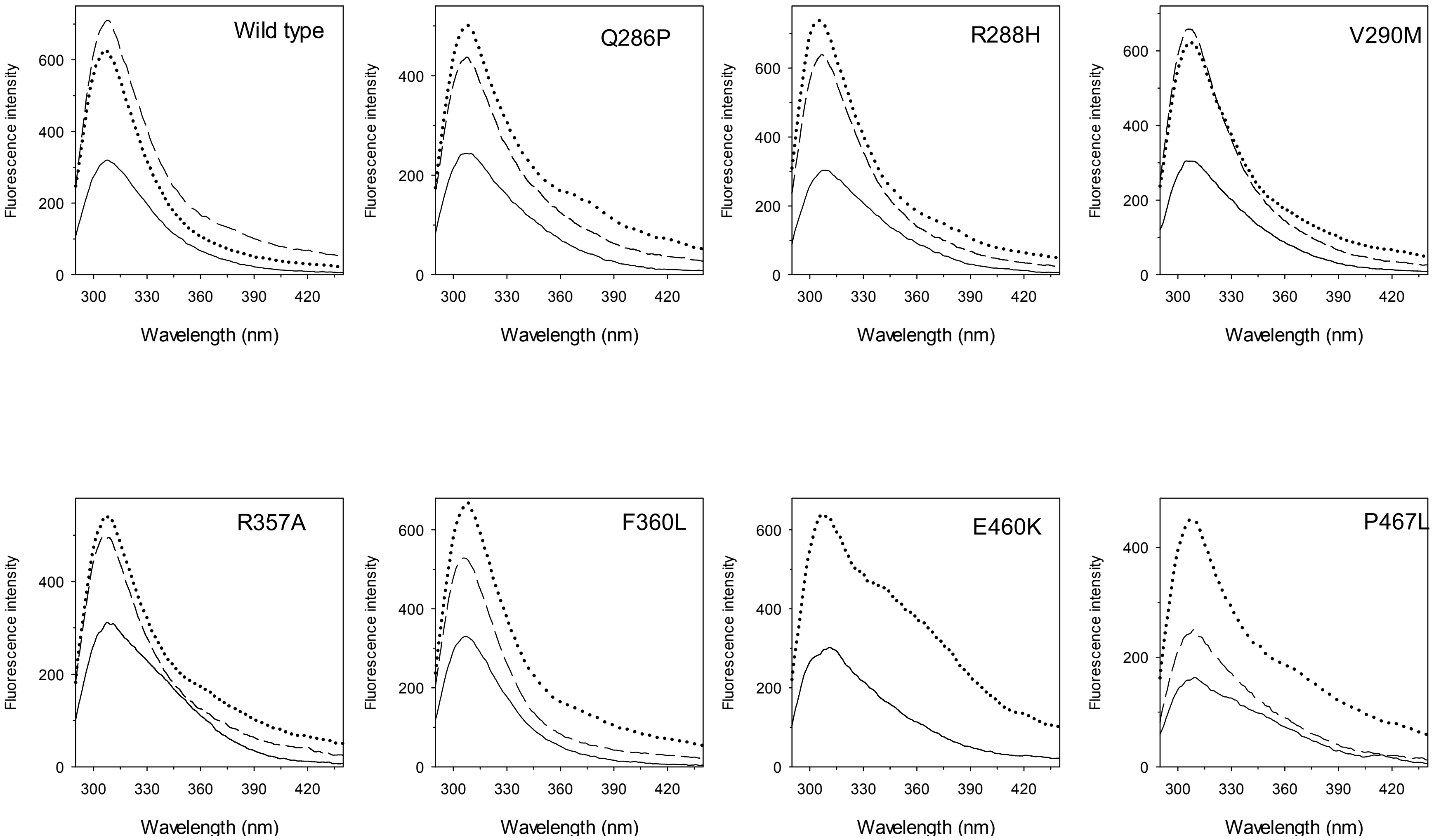

Figure S2. Intrinsic fluorescence emission spectra of PPARγ wild type and variants. Fluorescence spectra of PPARγ wild type and variants in 0 M (continuous lines), 8.0 M (dotted lines), 4.00 M (Q286P and F360L, dashed lines), and 4.07 M urea (wild type, R288H, V290M, and R357A, dashed lines) were recorded at 0.1 mg/mL protein concentration (274 nm excitation wavelength) in 20 mM Tris–HCl pH 8.0 containing 0.1 M NaCl and 0.2 mM DTT.

(**A**)

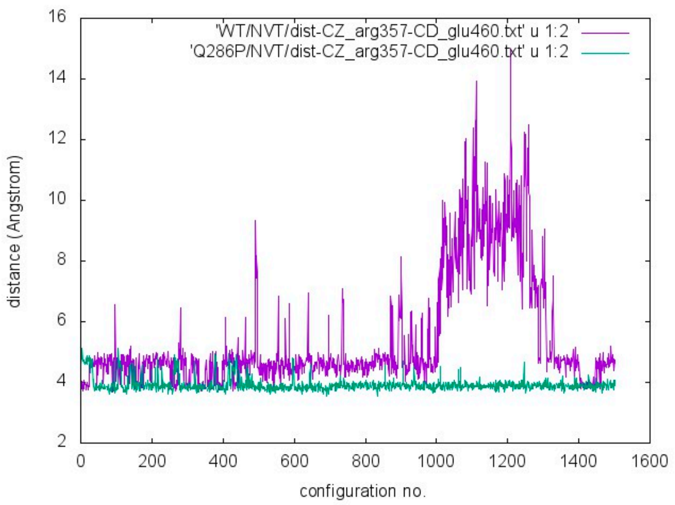

(**B**)

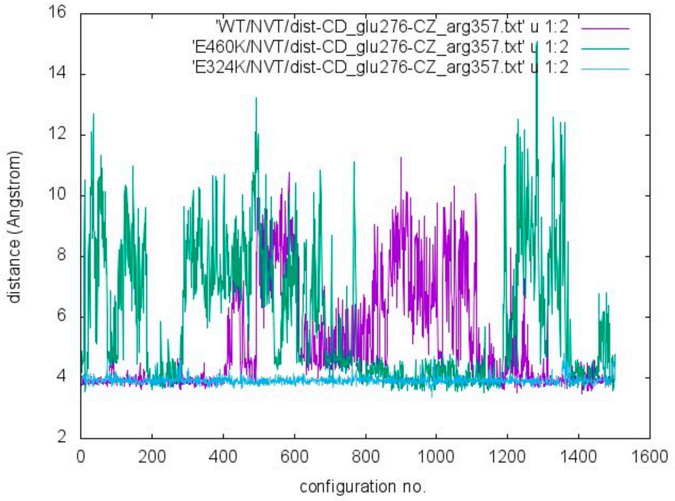

(**C**)

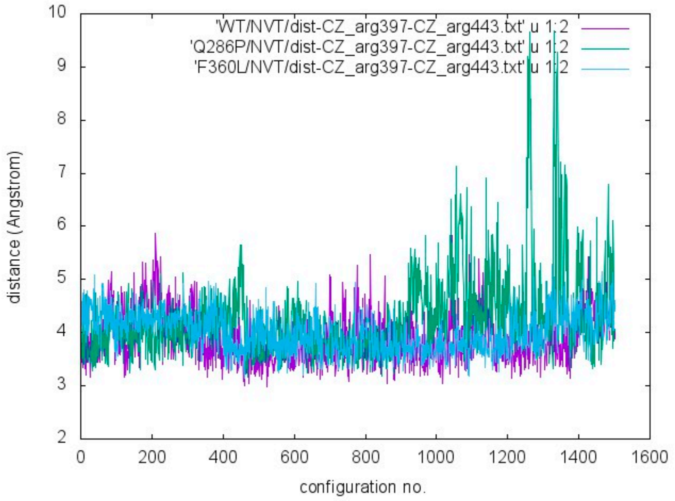

Figure S3. (**A**) Distance between R357 and E460. Distance between the carbon atom binding the two amino groups of arginine 357 lateral chain (CZ) and the carbon atom binding the carboxyl group of glutamic 460 lateral chain (CD), in the wild type and in the Q286P variant. On the *x*-axis is the configuration number; on the *y*-axis is the distance in Å; (**B**) Distance between E276 and R357. Distance between CD of glutamic 276 and CZ of arginine 357 in the wild type, in E460K, and in E324K variants. On the *x*-axis is the configuration number; on the *y*-axis is the distance in Å; (**C**) Distance between R397 and R443. Distance between CZ of arginine 397 and CZ of arginine 443 in the wild type, in Q286P, and in F360L variants. On the *x*-axis is the configuration number; on the *y*-axis is the distance in Å.

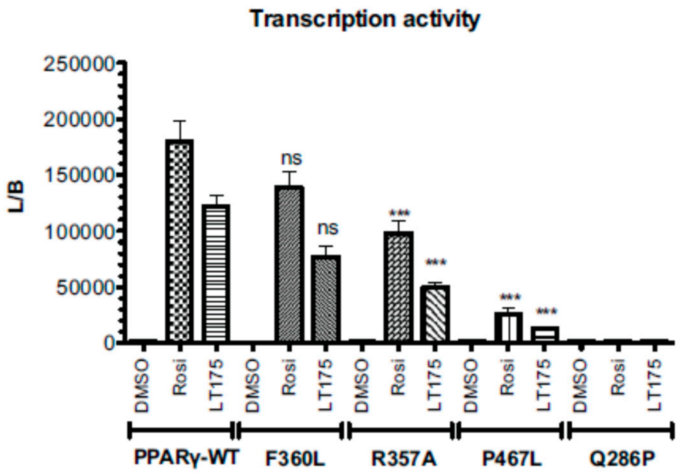

Figure S4. Transcription activity of wild type PPARγ and mutants in a PPARγ Gal4-based assay. Luciferase activity was normalized for difference in transfection efficiency by β-galactosidase activity and each point is the mean ± SEM of at least two experiments each performed in duplicate. Results are expressed as the ratio between luciferase activity and β-galactosidase activity (L/B), Differences between mutants and control (wild type with the same treatment) were significant (* $p < 0.05$; ** $p < 0.01$; *** $p < 0.001$; one-way ANOVA, Bonferroni test).

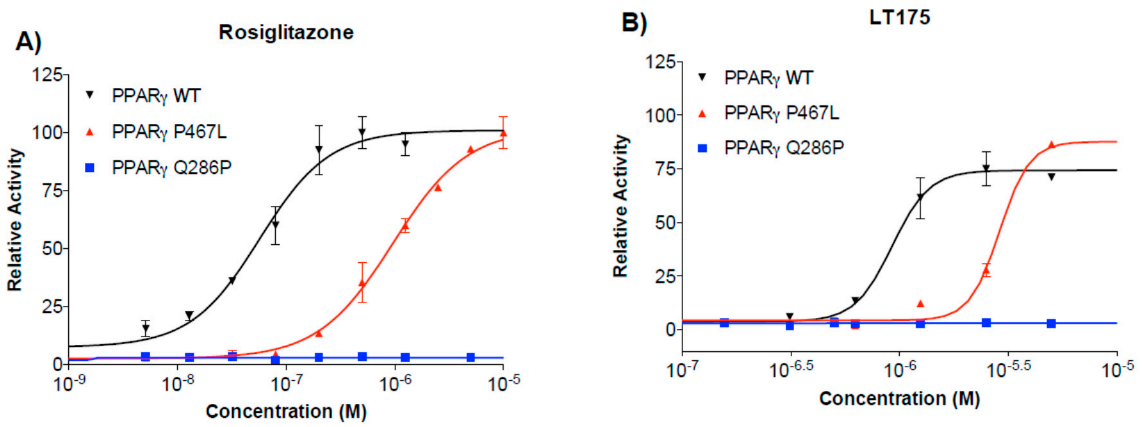

Figure S5. Transcription activity. Transcription activity of rosiglitazone (**A**); and LT175 (**B**) towards wild type PPARγ, PPARγ P467L, and PPARγ Q286P in a PPARγ Gal4-based assay. Results are expressed as a percentage of the highest efficacy obtained with rosiglitazone and each point is the mean ± SEM of two experiments each performed in duplicate.

Table S1. Transcription activity of PPARγ wild type and mutants in a PPARγ Gal4-based assay.

PPARγ	**Rosiglitazone**	**LT175**
PPARγ wild type	53 ± 17	1100 ± 180
PPARγ F360L	790 ± 70	2500 ± 500
PPARγ R357A	380 ± 70	1530 ± 270
PPARγ P467L	960 ± 180	2880 ± 290
PPARγ Q286P	-	-

Potency of rosiglitazone and LT175 towards wild type PPARγ and mutants in a PPARγ Gal4-based assay. Results are expressed as EC_{50} (nM) and are the mean ± SEM of two experiments each performed in duplicate.

Table S2. List of oligonucleotides used for site-directed mutagenesis.

PPARγ Variant	Oligonucleotide
Q286P	Forward: GCATCTTTCAGGGCTGCCCGTTTCGCTCCGTGGAG<br>Reverse: CTCCACGGAGCGAAACGGGCAGCCCTGAAAGATGC
R288H	Forward: CAGGGCTGCCAGTTTCATTCCGTGGAGGCTGTGC<br>Reverse: GCACAGCCTCCACGGAATGAAACTGGCAGCCCTG
V290M	Forward: GGGCTGCCAGTTTCGCTCCATGGAGGCTGTGCAGGAGATC<br>Reverse: GATCTCCTGCACAGCCTCCATGGAGCGAAACTGGCAGCCC
R357A	Forward: GGAGTTTCTAAAGAGCCTGGCAAAGCCTTTTGGTG<br>Reverse: CACCAAAAGGCTTTGCCAGGCTCTTTAGAAACTCC
F360L	Forward: GCCTGCGAAAGCCTCTGGGTGACTTTATGGAGCCC<br>Reverse: GGGCTCCATAAAGTCACCCAGAGGCTTTCGCAGGC
P467L	Forward: GACATGAGTCTTCACCTGCTCCTGCAGGAG<br>Reverse: CTCCTGCAGGAGCAGGTGAAGACTCATGTC
E460K	Forward: GCAGGTGATCAAGAAGACGAAGACAGACATGAGTCTTCACCCGC<br>Reverse: GCGGGTGAAGACTCATGTCTGTCTTCGTCTTCTTGATCACCTGC
R397C	Forward: AGTGGAGACTGCCCAGGTTTGCT<br>Reverse: AGCAAACCTGGGCAGTCTCCACT
E324K	Forward: CCTCAAATATGGAGTCCACAAGATCATTTACACAATGCTGGCC<br>Reverse: GGCCAGCATTGTGTAAATGATCTTGTGGACTCCATATTTGAGG

Table S3. PPARγ variants.

PPARγ Variant	SNP ID	Reference
Q286P	rs121909242	[2]
R288H	rs28936407	[2]
V290M	rs72551362	[3]
E324K	rs530007199 COSM1037602	https://bioinfo.uth.edu/TSGene/gene_mutation.cgi?gene=5468#cosmic [4];<br>http://pedican.bioinfo-minzhao.org/gene_mutation.cgi?gene=5468 [5]
R357A	–	[6]
F360L	rs72551363	[7]
R397C	rs72551364	[6]
E460K	–	https://bioinfo.uth.edu/TSGene/gene_mutation.cgi?gene=5468#cosmic [4];<br>http://pedican.bioinfo-minzhao.org/gene_mutation.cgi?gene=5468 [5]
P467L	rs121909244	[6,8]

References

1. Nolte, R.T.; Wisely, G.B.; Westin, S.; Cobb, J.E.; Lambert, M.H.; Kurokawa, R.; Rosenfeld, M.G.; Willson, T.M.; Glass, C.K.; Milburn, M.V. Ligand binding and co-activator assembly of the peroxisome proliferator-activated receptor-γ. *Nature* **1998**, *395*, 137–143.
2. Sarraf, P.; Mueller, E.; Smith, W.M.; Wright, H.M.; Kum, J.B.; Aaltonen, L.A.; de la Chapelle, A.; Spiegelman, B.M.; Eng, C. Loss-of-function mutations in PPARγ associated with human colon cancer. *Mol. Cell* **1999**, *3*, 799–804.
3. Puhl, A.; Webb, P.; Polikarpov, I. Structural dataset for the PPARγ V290M mutant. *Data Brief.* **2016**, *7*, 1430–1437.
4. Tumor Suppressor Gene Database. Available online: https://bioinfo.uth.edu/TSGene/gene_mutation.cgi?gene=5468#cosmic (accessed on 31 October 2016).
5. Pediatric Cancer Gene Database. Available online: http://pedican.bioinfo-minzhao.org/gene_mutation.cgi?gene=5468 (accessed on 31 October 2016).
6. Jeninga, E.H.; Gurnell, M.; Kalkhoven, E. Functional implications of genetic variation in human PPARγ. *Trends Endocrinol. Metab.* **2009**, *20*, 380–387.

7. Lori, C.; Pasquo, A.; Montanari, R.; Capelli, D.; Consalvi, V.; Chiaraluce, R.; Cervoni, L.; Loiodice, F.; Laghezza, A.; Aschi, M.; et al. Structural basis of the transactivation deficiency of the human PPARγ F360L mutant associated with familial partial lipodystrophy. *Acta. Crystallogr. D Biol. Crystallogr*. **2014**, *70*, 1965–1976.
8. Kallenberger, B.C.; Love, J.D.; Chatterjee, V.K.; Schwabe, J.W. A dynamic mechanism of nuclear receptor activation and its perturbation in a human disease. *Nat. Struct. Biol.* **2003**, *10*, 136–140.
